# Simulation of passive exotendon assistive device for agricultural harvesting task

**DOI:** 10.1007/s13246-023-01305-9

**Published:** 2023-07-26

**Authors:** Yon Sin Chan, Yu Xuan Teo, Darwin Gouwanda, Surya Girinatha Nurzaman, Alpha Agape Gopalai

**Affiliations:** 1 School of Engineering, Monash University Malaysia, Subang Jaya, Selangor Malaysia; 2 School of Engineering, Monash University Malaysia & Monash Industry Palm Oil Research Platform, Jalan Lagoon Selatan, Bandar Sunway, 47500 Subang Jaya, Selangor Malaysia

**Keywords:** Assistive device, Agricultural harvesting, Passive exoskeleton, Musculoskeletal simulation, Back muscles

## Abstract

This study proposes and investigates the feasibility of the passive assistive device to assist agricultural harvesting task and reduce the Musculoskeletal Disorder (MSD) risk of harvesters using computational musculoskeletal modelling and simulations. Several passive assistive devices comprised of elastic exotendon, which acts in parallel with different back muscles (rectus abdominis, longissimus, and iliocostalis), were designed and modelled. These passive assistive devices were integrated individually into the musculoskeletal model to provide passive support for the harvesting task. The muscle activation, muscle force, and joint moment were computed with biomechanical simulations for unassisted and assisted motions. The simulation results demonstrated that passive assistive devices reduced muscle activation, muscle force, and joint moment, particularly when the devices were attached to the iliocostalis and rectus abdominis. It was also discovered that assisting the longissimus muscle can alleviate the workload by distributing a portion of it to the rectus abdominis. The findings in this study support the feasibility of adopting passive assistive devices to reduce the MSD risk of the harvesters during agricultural harvesting. These findings can provide valuable insights to the engineers and designers of physical assistive devices on which muscle(s) to assist during agricultural harvesting.

## Introduction

The growing popularity of computational musculoskeletal modelling to replicate the kinematics and kinetics of real humans has piqued the interest of researchers worldwide. It saves researchers the expenses and time required to conduct numerous physical experiments such as device testing and user training. Aside from mimicking real human movement, the simulated model can generate biomechanical information that is difficult to detect directly with present motion sensors, including joint moment and muscle force. Furthermore, an assistive device can be modelled and simulated in the biomechanical model to provide insights and guidelines for designing and developing a physical wearable exoskeleton [1–3].

An exoskeleton is a wearable assistive device that augments, enables, assists or improves human movement, posture or physical activity. It can be broadly classified as an active and a passive exoskeleton. The active exoskeleton uses powered force or torque-producing components such as a motor to generate power. In contrast, the latter employs unpowered mechanisms such as springs and dampers to create force or torque [4]. The passive exoskeleton is lighter, less costly and easier to use because they do not need any electromechanical hardware. In the case of an active exoskeleton, battery capacity and charging time could adversely affect the efficiency of the activity. As a result, the passive exoskeleton is preferred for the occupational environment [5].

Despite the increasing adoption in industrial applications [6], the development and use of exoskeletons in agriculture are still in infancy. Until 2021, there have only been approximately eight studies that employ exoskeletons for agricultural tasks [7]. According to a study by *Upasani et al.* [8], the exoskeleton may have a greater influence in the agricultural field than in the industrial sector owing to the dynamic nature of the activity, which restricts the adoption of other intervention strategies necessitating significant work environment modification. For instance, farm-friendly and practical exoskeletons might allow farmers with mild to moderate mobility issues to work without requiring significant changes to their work equipment [8].

One of the agricultural fields where the assistive device can be beneficial is oil palm Fresh Fruit Bunch (FFB) harvesting. Oil palm production has increased tremendously from 1.48 million tonnes in 1961 to 71.45 million tonnes in 2018 [9]. Despite the growing demand for palm oil worldwide, harvesters still manually handle oil palm harvesting. The harvester must visually recognize and make multiple tugging motions to harvest the ripe FFB. Given that the harvesters must continually retain awkward postures throughout the day to complete their harvesting task and that their salary is based on their daily output, they are highly exposed to a significant risk of Musculoskeletal Disorder (MSD), such as low back pain [10].

Numerous extant studies employ surveys [10–12], interviews [12, 13] and direct observation assessments [13, 14] to investigate harvesting activity. These studies establish the relationship between MSD risk and harvesting activity, but they do not offer guidance to effectively assist the harvesters. To the best of our knowledge, there has been only one study [7] investigating the viability of assistive devices for FFB harvesting. To date, an assistive device with musculoskeletal modelling and simulation approach has not been explored, developed and evaluated. Hence, this study aims to investigate the passive assistive device that can assist oil palm harvesting and reduce the MSD risk of the harvesters using musculoskeletal modelling and simulation. The findings of this study could shed light on the design and development of a physical assistive device that can assist the affected muscle(s), enhance the harvester’s posture and minimize the risk of injury.

## Methodology

Kinematics data must be captured and imported into the musculoskeletal model to generate the output motion. Motion Capture system (MoCap) is the “gold standard” in measuring joint kinematics, but it has its inherent limitations. The acquisition space is limited. Several cameras must be placed all around the area to record the motion. Hence, it is best suited for a fixed environment, like a laboratory and impractical for outdoor activities [15]. On the other hand, the Inertial Measurement Unit (IMU) is cheaper, lighter, less prone to marker occlusion and can produce results on par with MoCap [16]. Hence, IMU was selected for this study.

Six experienced male harvesters who were right-handed (Age: 33.5 ± 6.0 years old, Height: 168.83 ± 4.74 cm, Weight: 56.83 ± 4.26 kg) were recruited at the Malaysia oil palm plantation. They were informed about the experiment’s methodology and signed written consent prior to the experiment. This study was evaluated and approved by Monash University Human Research Ethics Committee. Six IMU sensors were attached to the harvester’s sternum, lumbar, upper arms, and wrists based on the recommendation of the commercial motion capture software Moveo Explorer. The sampling frequency was set at 1000 Hz. The harvesters were instructed to stand upright and stay still for three seconds for sensor calibration. They then harvested the trees with a height between 3 and 5 m for a minute. It was repeated three times for each harvester. The experimental data used in this study and the data reported in our previous work [17] are the same data. The raw data collected during the experiment include the 3D acceleration and 3D angular velocity of the sternum, lumbar, left and right upper arms, and left and right wrist.

The harvesting process is laborious. The harvester must manoeuvre around the tree to locate the ripe FFB and remove any fronds that might obstruct the harvesting. The harvester then places the sickle on the FFB stalk and cuts the stalk by tugging the sickle downwards. Multiple pulling motions are usually required to harvest the fruit. For a more accurate and representative analysis, the IMU data of all the pulling motions for each trial were identified and extracted for each harvester. Since these data may have different durations, they were linearly interpolated. Subsequently, a single pulling/harvesting motion was created by averaging all these motions in a single trial for each harvester.

**Fig. 1:**
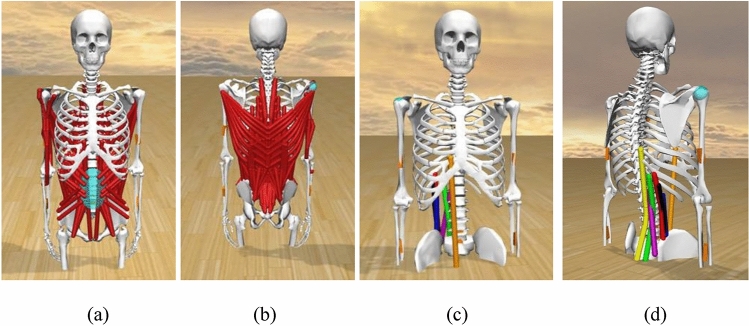
Harvesting musculoskeletal model **a** from front view, **b** from posterior view, **c** with passive assistive devices from front view, **d** with passive assistive devices from posterior view (Orange: Rectus abdominis, Yellow: Longissimus T9, Green: Longissimus R11, Purple: Longissimus L1, Blue: Iliocostalis R12, Black: Iliocostalis R11, Red: Iliocostalis 10). The muscles (highlighted in red in Fig. 1a and Fig. 1b) were hidden in Fig. 1c and Fig. 1d) for better view of the passive assistive devices

An OpenSim musculoskeletal model reported in [17] was adopted in this study, as depicted in Fig. 1a and b. This model contains 30 body segments, 238 musculotendon actuators and ten upper-extremity Hill-type muscle groups: erector spinae (iliocostalis and longissimus), latissimus dorsi, quadratus lumborum, external obliques, internal obliques, psoas major, rectus abdominis, multifidus, triceps, and biceps. The torso was modelled using the thoracic and cervical portions of the spine, the ribcage, the head, and the scapula, with spherical joints between six intervertebral joints. Through linear kinematic coordinate coupling restrictions, the vertebrae follow a spinal rhythm that distributes the trunk motion throughout these six intervertebral joints [17].

The harvesting tool was weighed, and the pulling/cutting force during harvesting was acquired from [18]. An external force of 164 N was applied to each hand of the simulated harvester, assuming that the harvesting force of 328 N was distributed equally between the two hands. This study added reserve actuators with an optimal force of 30 N to the back (lumbosacral joint: L5/S1), shoulder, and elbow joints [17]. A reserve actuator is a supplementary force or moment that accounts for the action of ignored passive structures [19], such as ligaments, capsular tissue, fascia, and cartilage. This step is essential to produce more accurate findings because musculoskeletal model presumes the active muscles are the only contributor to joint motion. In reality, passive joint moments (i.e., the joint moment created by the passive structures) also contribute to joint motion [20]. The peak reserve actuator moments were normalized to the peak net joint moments and then averaged across all trials. The average normalized peak reserve actuator moments of different joint motions were acquired in our earlier study [17]. The back lateral bending, shoulder flexion-extension, shoulder abduction-adduction, shoulder rotation and elbow flexion–extension had average normalized peak reserve actuator moments of less than 5%. The back rotation had an average normalized peak reserve actuator moment of 5.56%, and the highest value was found at the back flexion-extension, at 8.80%.

The Inverse Kinematics tool in OpenSim was used to iteratively move the model into a position that best matches the experimental sensor orientations by minimizing a sum of weighted squared orientation errors for each time frame of the experimental data. The raw data measured by the IMUs were initially processed using a 4th-order Butterworth low-pass filter with a cut-off frequency of 6 Hz. They were then converted to data that are compatible with OpenSim using a code written in MATLAB. These data were used as the input to generate the output motion [21]. Inverse Dynamics then used the model’s known motion to solve the equation of motion for the unknown generalized forces, as described in (1) to determine the joint moment [21].

1 $$M\left(q \right)\mathop {{\text{ }}\ddot{q}}\limits^{{}} + C\left( {q,\dot{q}} \right) + G\left( q \right) = {\mkern 1mu} \tau$$

where *q*, $\dot{q}$ and $\ddot{q}$ refer to the vector of generalized positions, velocities, and accelerations, respectively. *M(q)* is the system mass matrix, $C\left(q, \dot{q}\right)$ is the vector of Coriolis and centrifugal forces, *G*(*q*) is the vector of gravitational forces, and $\tau$ is the vector of generalized forces.

In the next step, Static Optimization was performed to estimate the muscle activation and muscle force. Static Optimization is the extension of Inverse Dynamics that transforms the net joint moment to a single muscle force at each instant. The muscle force was resolved by minimizing the total squared muscle activation, as indicated in (2) [21]. The ideal force generators were bounded by force-length-velocity characteristics, as described in (3).

2 $$\mathop \sum \limits_{{m = 1}}^{n} a_{m} ^{2}$$

3 $$\sum\limits_{{m = 1}}^{n} {\left[ {a_{m} f\left( {F_{m}^{0} ,\,l_{m} ,\,v_{m} } \right)} \right]} r_{{m,~j}} = ~\tau _{j}$$

where *n* is the number of muscles in the model, ${a}_{m}$ is the activation level of muscle m at each instant, ${F}_{m}^{0}$ is the maximum isometric force, ${l}_{m}$ is the length, ${v}_{m}$ is the shortening velocity, $f\left({F}_{m}^{0}, {l}_{m}, {v}_{m}\right)$is the force-length-velocity surface, ${r}_{m, j}$is the moment arm about the *j*th joint axis, and ${\tau }_{j}$is the generalized force acting about the *j*th joint axis.

The Inverse Kinematics, Inverse Dynamics and Static Optimization were first performed sequentially on the unassisted musculoskeletal models of six harvesters. It was found that rectus abdominis, longissimus L1, R11, and T9, and iliocostalis R10, R11 and R12 had the highest muscle activation during harvesting. Therefore, this study designed and evaluated seven passive assistive devices, as outlined in Fig. 1c and d. Each device comprised of one elastic exotendon that was integrated individually—parallel to the muscle: rectus abdominis, longissimus L1, R11 and T9, and iliocostalis R10, R11 and R12. Only one assistive device was investigated at one time in one simulation. The elastic exotendon was modelled as a massless force element applied to each muscle, and the tension of the exotendon is defined in (4) [22].

4 $$T = K~ \times s \times \left( {1 + D\dot{L}} \right)$$

where *T* is the exotendon’s tension, *K* is the exotendon’s stiffness, *s* is the exotendon’s stretch length (*s* is zero when *L* is less than or equal to *L*_*o*_, whereas *s* is the difference between *L* and *L*_*o*_ when *L* is greater than *L*_*o*_), *D* is the dissipation factor, *L* is the exotendon’s path length, *L*_*o*_ is the exotendon’s resting length, and $\dot{L}$ is the derivative of the exotendon’s path length. The exotendon dissipation factor was set at 0.01s/m [23].

The resting length and stiffness of the exotendon are two main factors that affect the performance of the assistive device [24]. Considering that the simulation is computationally intensive and time-consuming, particularly when there are three trials for each subject, and with seven assistive devices to run individually, only three harvesters were investigated to identify the optimal resting length and stiffness.

- Resting length: Two different resting lengths were considered. The first resting length is defined as a combination of the optimal fibre length and tendon slack length, as depicted in (5) [25]. Tendon generates force at tendon slack length, whereas muscle fibre produces active force at 40% of its optimal fibre length.

5 $$L_{{r,1}} = 0.4~L_{o}^{M} \, + \,L_{s}^{T}$$

where ${L}_{r,1}$ is the exotendon’s resting length, ${L}_{o}^{M}$ is the optimal fibre length of the assisted muscle, and ${L}_{s}^{T }$is the tendon slack length of the assisted muscle. The second resting length is defined as the optimal fibre length of the assisted muscle and denoted as L_r,2_ (i.e. L_r,2_ = ${\text{L}}_{\text{o}}^{\text{M}}$). This value was selected because muscle begins to generate passive force at its optimal fibre length [26]. The values of L_r,1_ and L_r,2_ for each assistive device are presented in Table 1. The stiffness of the exotendon was set at 10kN/m in both cases.

**Table 1:** The first and second resting lengths (L_r,1_ and L_r,2_) for the assistive device

Assistive device	L_r,1_ (m)	L_r,2_ (m)
Rectus abdominis	0.20	0.30
Iliocostalis R10	0.16	0.16
Iliocostalis R11	0.12	0.14
Iliocostalis R12	0.09	0.10
Longissimus L1	0.14	0.09
Longissimus R11	0.21	0.12
Longissimus T9	0.28	0.14

- Stiffness: A range of exotendon stiffness between 1kN/m and 10kN/m was commonly used in previous studies [27]. Hence, exotendon stiffness of 1kN/m and 10kN/m were adopted and denoted as K_1_ and K_2_, respectively. The resting length of the exotendon was set at L_r,2_ in both cases.

In order to identify the optimal resting length and stiffness, the selection process focuses on the assistive device that minimizes the muscle activation and muscle force. This ensures the assistive device’s effectiveness in enhancing support during harvesting. These two parameters were then used in the Inverse Dynamics of the assisted musculoskeletal models of six harvesters to determine the joint moment. The peak joint moments found in all trials were averaged for each harvester. They were then normalized to each harvester’s body weight and averaged over harvesters. Next, Static Optimization was performed on the same models to compute the muscle activation and muscle force. The peak muscle activation and peak muscle force for each trial were normalized to the maximum values of each harvester and averaged across all trials. The individual muscle fascicle activations were aggregated by summation to obtain the overall muscle activation. For instance, the simulated activation level of the longissimus muscle was computed by summing the activations of its lumbar and thoracic components within the longissimus thoracis. This method was similarly employed for the iliocostalis muscle. The Inverse Dynamics and Static Optimization did not change the kinematics of the upper extremity. The use of exotendon was expected to provide necessary assistance by reducing the joint moment, muscle activation and/or muscle force.

One-way ANOVA (Analysis of Variance) was conducted to examine the significant difference in normalized peak joint moment, muscle activation and muscle force between unassisted and assisted motions. Tukey-kramer multiple comparison test was performed if the significant difference was found between them. The significance level was set at 0.05.

## Result

The reduction in average peak muscle activation and muscle force for the assistive devices with variable resting lengths is presented in Table 2. Almost all of the assisted motions exhibited reduced muscle activation and muscle force at longissimus and iliocostalis when the assistive devices were placed at Longissimus T9, L1, and R11 and when L_r,2_ was used. The assistive devices showed variable effects on the muscle activation and muscle force of the rectus abdominis. An increase in peak muscle activation and muscle force were found at the rectus abdominis when the assistive devices were placed along the longissimus muscle. Nevertheless, L_r,2_ revealed better results in reducing the muscle activation and muscle force for most muscles under all assisted conditions; hence it was selected in this study.

**Table 2:** Reduction in average peak muscle activation and muscle force (n = 6) for the assistive devices with variable resting lengths (Positive value indicates a decrease in the muscle activation or muscle force and vice versa)

Assistive device	Reduction in the average peak muscle activation		Reduction in the average peak muscle force	
	L_r,1_	L_r,2_	L_r,1_	L_r,2_
Longissimus				
Rectus abdominis	− 3.32%	20.07%	− 107.00%	32.03%
Iliocostalis R10	24.46%	24.46%	44.79%	44.79%
Iliocostalis R11	22.99%	23.24%	43.43%	41.41%
Iliocostalis R12	24.06%	22.99%	43.49%	41.19%
Longissimus T9	25.15%	49.22%	47.05%	82.00%
Longissimus L1	24.16%	30.71%	45.74%	67.14%
Longissimus R11	4.16%	42.74%	44.76%	70.47%
Iliocostalis				
Rectus abdominis	11.81%	15.23%	− 61.22%	30.93%
Iliocostalis R10	20.84%	20.84%	49.36%	49.36%
Iliocostalis R11	19.19%	18.65%	46.27%	43.49%
Iliocostalis R12	19.90%	18.42%	47.95%	43.16%
Longissimus T9	18.71%	39.71%	45.81%	78.67%
Longissimus L1	18.66%	31.99%	47.03%	70.06%
Longissimus R11	− 10.73%	33.30%	45.70%	74.29%
Rectus abdominis				
Rectus abdominis	40.07%	8.37%	68.56%	13.26%
Iliocostalis R10	6.17%	6.17%	7.70%	7.70%
Iliocostalis R11	7.74%	7.31%	9.50%	9.46%
Iliocostalis R12	6.80%	7.46%	9.59%	9.86%
Longissimus T9	2.40%	− 7.74%	− 0.73%	− 97.11%
Longissimus L1	4.09%	13.06%	3.11%	− 26.69%
Longissimus R11	− 22.30%	− 6.39%	4.80%	− 46.11%

Table 3 summarizes the reduction in average peak muscle activation and muscle force for the assistive devices with variable exotendon stiffness. Assistive devices with stiffness of K_2_ produced lower muscle activation and muscle force for the longissimus and iliocostalis muscle than the devices with stiffness of K_1_. Substantial reductions can be observed when the devices were placed along the longissimus T9, L1, and R11. However, these devices produced opposing effects on the rectus abdominis. A large increase in rectus abdominis was found, particularly when assistance was applied along the longissimus T9. Overall, K_2_ displayed better results in reducing muscle activation and muscle force for most muscles under all assisted conditions; hence it was utilized in this study.

**Table 3:** Reduction in average peak muscle activation and muscle force (n = 6) for the assistive devices with variable exotendon stiffness (Positive value indicates a decrease in the muscle activation or muscle force and vice versa)

Assisted device	Reduction in the average peak muscle activation		Reduction in the average peak muscle force	
	K_1_	K_2_	K_1_	K_2_
Longissimus				
Rectus abdominis	11.12%	20.07%	30.09%	32.03%
Iliocostalis R10	21.44%	24.46%	39.71%	44.79%
Iliocostalis R11	21.30%	23.24%	39.34%	41.41%
Iliocostalis R12	21.26%	22.99%	39.31%	41.19%
Longissimus T9	25.61%	49.22%	46.55%	82.00%
Longissimus L1	22.90%	30.71%	42.55%	67.14%
Longissimus R11	23.66%	42.74%	44.42%	70.47%
Iliocostalis				
Rectus abdominis	10.97%	15.23%	19.96%	30.93%
Iliocostalis R10	15.96%	20.84%	38.95%	49.36%
Iliocostalis R11	15.99%	18.65%	38.23%	43.49%
Iliocostalis R12	15.98%	18.42%	38.17%	43.16%
Longissimus T9	20.10%	39.71%	44.77%	78.67%
Longissimus L1	18.11%	31.99%	41.98%	70.06%
Longissimus R11	9.17%	33.30%	44.08%	74.29%
Rectus abdominis				
Rectus abdominis	− 2.05%	8.37%	12.90%	13.26%
Iliocostalis R10	7.65%	6.17%	9.94%	7.70%
Iliocostalis R11	7.78%	7.31%	10.13%	9.46%
Iliocostalis R12	7.79%	7.46%	10.16%	9.86%
Longissimus T9	4.00%	− 7.74%	0.35%	− 97.11%
Longissimus L1	6.55%	13.06%	6.92%	− 26.69%
Longissimus R11	4.21%	− 6.39%	5.56%	− 46.11%

**Fig. 2:**
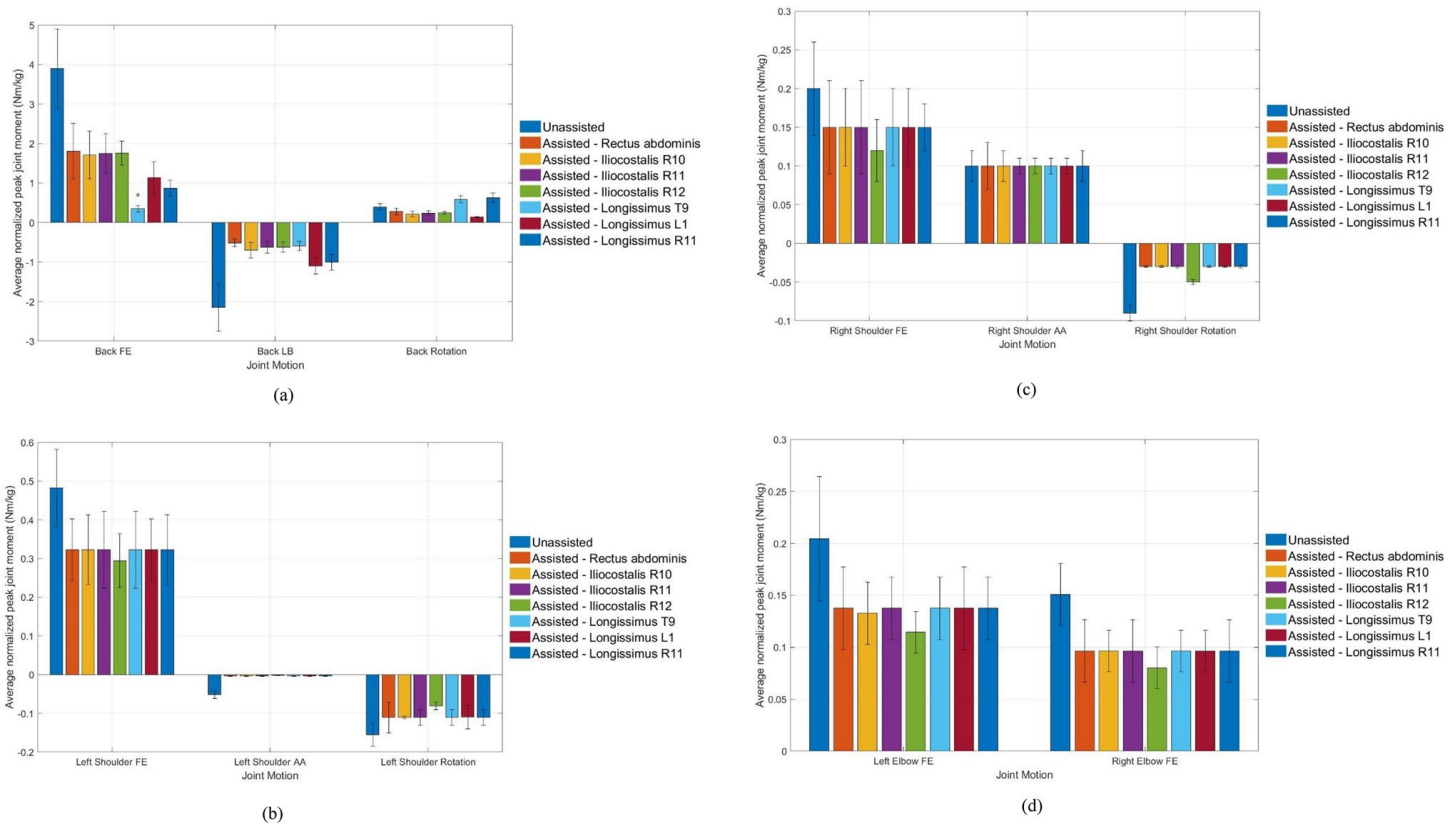
Average normalized peak joint moment (n = 6) of the unassisted and assisted motions for the **a** back, **b** left shoulder, **c** right shoulder and **d** elbow joints (*FE* flexion–extension, *LB* lateral bending, *AA* adduction–abduction; Positive values represent flexion, lateral bending to the right, adduction, and rotation to the right; negative values represent extension, lateral bending to the left, abduction, and rotation to the left)

The average normalized peak joint moment of the unassisted and assisted motions for the back, shoulder and elbow joints is depicted in Fig. 2. Peak joint moment during all assisted motions was significantly lower than those during the unassisted motion for back flexion and lateral bending. The back flexion peak joint moment reduced from 3.90 ± 1.01Nm/kg to a range of 0.35 ± 0.08 and 1.81 ± 0.73, corresponding to a reduction of between 53.54 and 91.08%. Significant reduction was found when the longissimus T9 muscle was assisted. Peak joint moment of back lateral bending decreased from 2.15 ± 0.66Nm/kg to a range of 0.51 ± 0.10 and 1.10 ± 0.19Nm/kg, representing a decrease of between 49.06 and 76.39%. On the other hand, the peak joint moment for the back rotation exhibited a reduction during all assisted motions except the longissimus T9 and R11 assistive devices, which increased the peak joint moment. All assistive devices decreased the joint moment for shoulder and elbow motions compared to the non− assisted cases, except for the constant joint moment in right shoulder adduction. Hence, it can be deduced that placing assistive device along the rectus abdominis and iliocostalis muscles is a better alternative in reducing the joint moment rather than placing it at the longissimus, particularly the T9 and R11 fascicles.

**Fig. 3:**
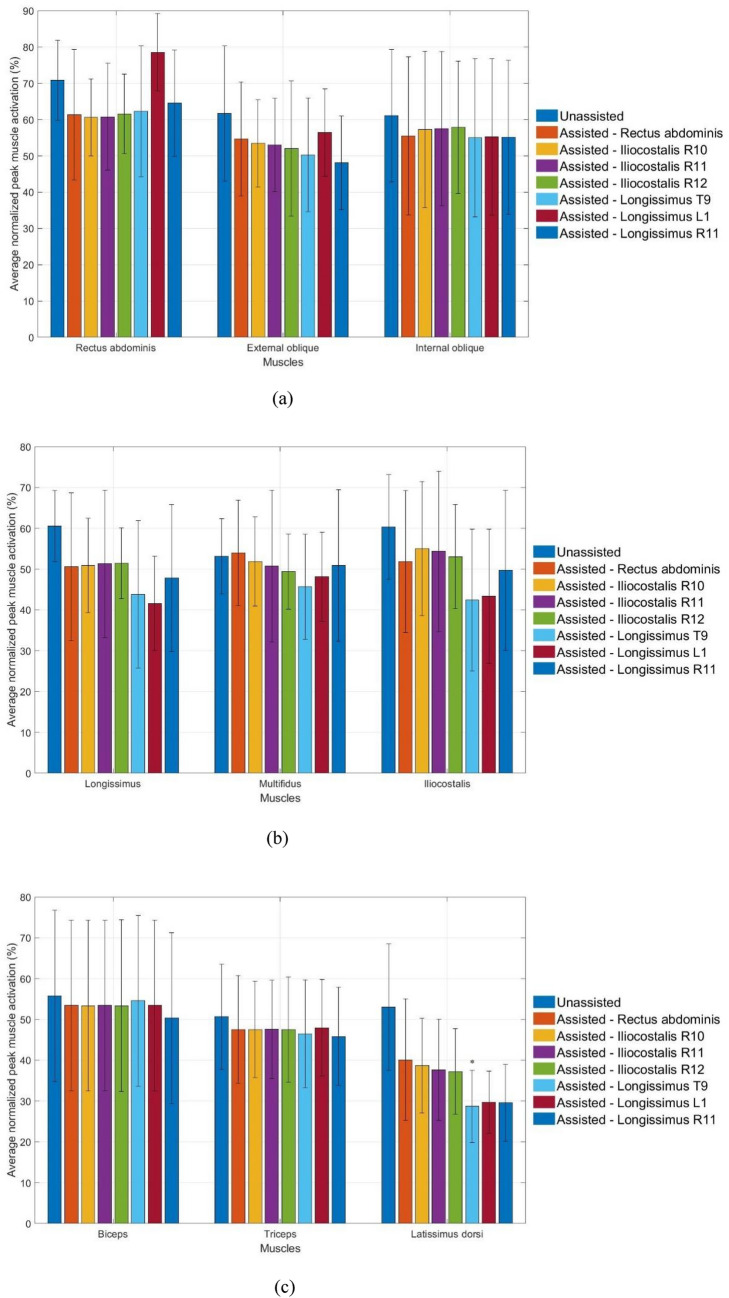
Average peak normalized muscle activation (n = 6) of the unassisted and assisted simulated harvesters for **a** rectus abdominis, external oblique, internal oblique, **b** longissimus, multifidus, iliocostalis, **c** biceps, triceps and latissimus dorsi

Figure 3 illustrates the average normalized peak muscle activation of the unassisted and assisted simulated harvesters. Considerable reduction in the peak activation of the latissimus dorsi can be observed for all assisted motions. Their peak muscle activation decreased from 53.02 ± 15.47% to between 28.72 ± 8.89 and 40.12 ± 14.89%, corresponding to a reduction of between 24.34% and 45.83%. The peak activation of the longissimus was reduced from 60.57 ± 8.72% to between 50.00 ± 11.55 and 51.50 ± 8.72% for the rectus abdominis and iliocostalis assistive devices, and a range of between 41.50 ± 11.55 and 44.00 ± 18.11% for the longissimus assistive devices. Generally, the peak activation of all muscles decreased for all assistive devices except the multifidus and rectus abdominis when rectus abdominis and longissimus T9 fascicle were assisted, respectively.

**Fig. 4:**
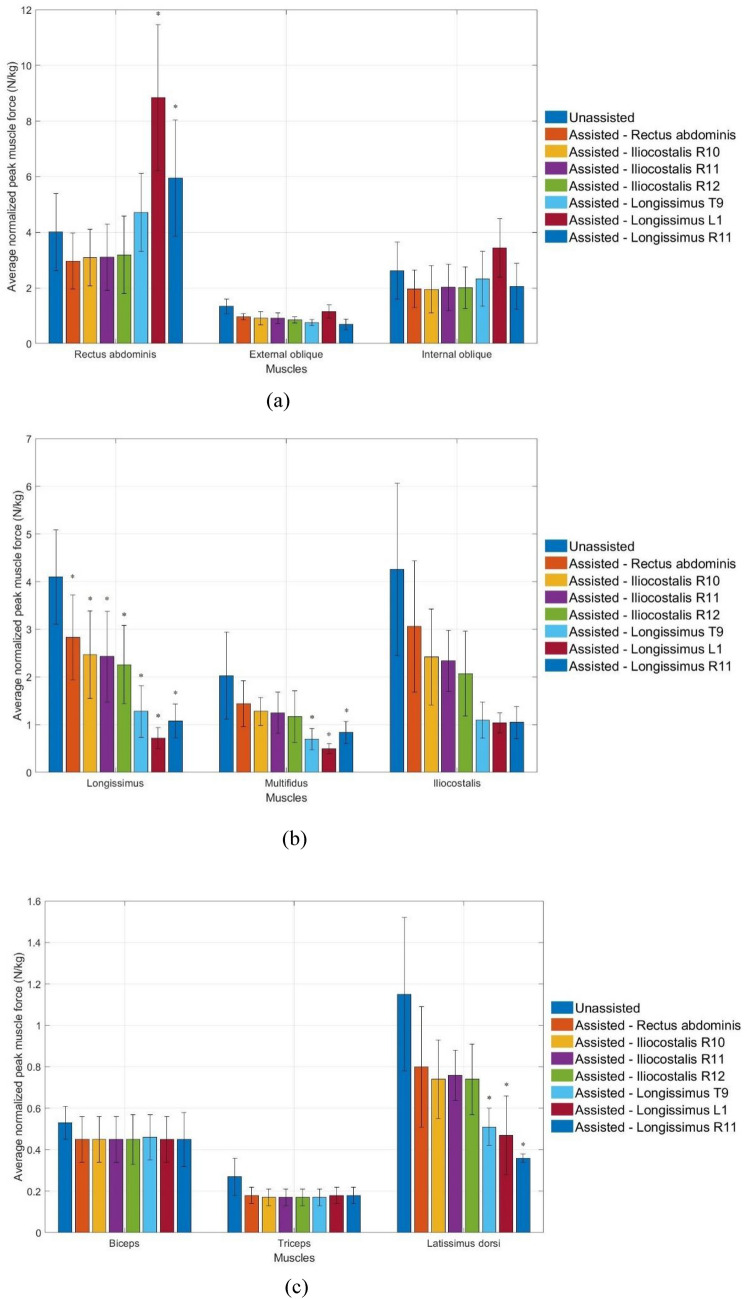
Average peak normalized muscle force (n = 6) of the unassisted and assisted simulated harvesters for **a** rectus abdominis, external oblique, internal oblique, **b** longissimus, multifidus, iliocostalis, **c** biceps, triceps and latissimus dorsi

Figure 4 displays the average normalized peak muscle force of the unassisted and assisted simulated harvesters. Since the longissimus, rectus abdominis, and iliocostalis demonstrated significant muscle force during unassisted motion, the muscle force of these muscles during assisted motion was evaluated. All assistive devices significantly reduce the peak muscle force of the longissimus (Unassisted: 4.10 ± 0.99 N/kg) and iliocostalis (Unassisted: 4.26 ± 1.81 N/kg) in the ascending order: rectus abdominis (2.83 ± 0.89 N/kg, 3.06 ± 1.38 N/kg), iliocostalis R12 fascicle (2.47 ± 0.92 N/kg, 2.42 ± 1.01 N/kg), iliocostalis R11 fascicle (2.43 ± 0.95 N/kg, 2.34 ± 0.64 N/kg), iliocostalis R10 fascicle (2.26 ± 0.82 N/kg, 2.07 ± 0.89 N/kg), longissimus L1 fascicle (1.28 ± 0.54 N/kg, 1.10 ± 0.38 N/kg), longissimus R11 fascicle (1.08 ± 0.35 N/kg, 1.05 ± 0.34 N/kg), and longissimus T9 fascicle (0.72 ± 0.22 N/kg, 1.04 ± 0.21 N/kg). The longissimus assistive devices increased the rectus abdominis muscle force, whereas its muscle force was reduced by the rectus abdominis and iliocostalis assistive devices. In summary, longissimus assistive devices significantly reduced the muscle force of most muscles by transmitting the loads to the rectus abdominis, indicating that rectus abdominis and iliocostalis assistive devices are more pragmatic in decreasing muscle force.

## Discussion

This study proposed the use of passive assistive device to assist the oil palm harvesting task and to reduce the risk of MSD. Several devices were developed to help and support the rectus abdominis, longissimus, and iliocostalis during harvesting. These muscles were selected because they are exposed to a higher risk of MSD during FFB harvesting [17]. The harvesting musculoskeletal model was adopted from our earlier work [17]. In contrast to many contemporary musculoskeletal models [28, 29] that assume the trunk as a rigid body, the harvesting musculoskeletal model was constructed using several rigid body segments, including the pelvis, each lumbar vertebrae, and torso. Since the trunk of a real human is made of a chain of interconnected vertebrae, scapula, and pelvis, the modelling of the trunk in the harvesting musculoskeletal model is more authentic [17]. Moreover, the simulated joint angle and muscle activation during harvesting were validated with the commercial motion capture software—Moveo Explorer and surface Electromyography (sEMG), respectively. Good correlations between simulated results and field measurements were reported in [17], demonstrating that the musculoskeletal model can simulate biomechanical parameters close to the real harvester.

The application of a passive exoskeleton to assist in harvesting motion was first reported in [7]. The authors examined three upper extremity muscles—biceps, anterior deltoid, and upper trapezius using sEMG in ten subjects without harvesting experience. The biceps muscle activation was reduced by 30.23% after wearing the exoskeleton. This result was much higher than our study, which ranged between 2.16% and 9.71%. This disparity was reasonable. Firstly, our study recruited experienced harvesters to ensure the simulated exoskeleton could assist the harvesting activity at the plantation. Secondly, the exoskeleton in our study was designed to assist the back muscles because of the prevalence of MSD at the harvesters’ lower back [13]. In contrast, the prototype exoskeleton in [7] was designed to assist arm raising. Despite the significant reduction found in muscle activation, the authors in [7] found that the prototype exoskeleton must be modified for better adoption. They attributed the lack of promising results to their incomplete grasp of the exoskeleton’s impact on the harvesters. The length of the harvesting time and frequency of repetition in this study may not be a suitable approximation of on-site harvesting too. Nevertheless, their results are encouraging because they indicate the need for a comprehensive investigation of the assistive device for agricultural harvesting.

Considering that limited studies analyzed harvesting activity using quantitative approaches and that the harvesting motion involved pulling motion, this work was compared with studies involving the pulling task. Nine male volunteers participated in *Lett* and *McGill*’s investigation [30] to examine the horizontal pulling task at two handle heights (set to shoulder and waist height) with three loads (44.5 N, 222.4 and 400.5 N) using a pulley system. The peak lumbar moment while pulling at shoulder height was equivalent to 1.62Nm/kg and 2.21Nm/kg when the load was 222.5 and 400.5 N, respectively. In another horizontal pulling study with a pulling force between 208.5 and 326.4 N involving twelve male university-aged participants [31], the average flexor torque measured by the researchers was equivalent to 0.48Nm/kg at the left shoulder joint and 1.67Nm/kg at the L5/S1 joint. Both studies used the segment link model to estimate the joint moment. The flexor moment of the left shoulder joint found in [31] is in line with this study’s finding, which discovered 0.48Nm/kg of the peak flexor moment for the same joint during unassisted harvesting motion. During unassisted harvesting motion, the peak flexor moment of the L5/S1 joint was found to be 3.90Nm/kg, which was higher than the values from the two studies [30, 31]. Since the pulling force is similar among these studies, the discrepancy may be due to the different elevation angles of the pulling motion. According to *McFarland et al.* [32]., the muscle demand increases with the elevation angle, indicating that the joint with higher elevation is subjected to a greater torque. *Syuaib* [33] used a video recording to estimate the elevation angle during FFB harvesting, and it was found to be at most 64°, much higher than 0° in the horizontal pulling. Hence, it is reasonable that the joint moment found in this study is higher than those with horizontal pulling.

This study discovered significant simulated muscle activation in the erector spinae (longissimus and iliocostalis) and abdominal muscles (rectus abdominis, internal oblique, and external oblique) during harvesting. These results are consistent with other studies on pulling tasks such as [34, 35] that utilized sEMG to examine muscle activation and found that the erector spinae had high activation. However, no discernible abdominal muscle activation was found. It might be due to the intrinsic limitations of sEMG. It is well known that the fatty tissue that typically covers these muscles can attenuate the sEMG signal, suggesting that the measured activations measured would be lower than the actual one [36]. *Lazzaroni et al.* [34] also agreed that the muscle activation would not be consistent among the studies because of the variation in experiment designs and limits. The lumbar load can differ considerably among participants owing to their individual variations.

Albeit not directly comparable, this study’s simulation results are congruent with prior studies involving various upper extremity activities [37–39]. The adoption of the simulated assistive devices with the musculoskeletal tool to assist the overhead motion [39] and FE of the back [38], elbow, and shoulder [37] significantly reduced the joint moment of the shoulder and elbow, muscle force of the erector spinae and biceps, respectively. The muscle force of the erector spinae with a simulated assistive device in [38] ranged from 143 to 371 N during back flexion. Their result is close to this study, which acquired 94.54–278.47 N in the total muscle force of the longissimus and iliocostalis. A recent systematic review [40] investigated the industrial back-support exoskeletons, focusing on lifting and bending activities. It discovered that the back muscle activation and L5/S1 joint moment were generally decreased in most studies. The erector spinae muscle activation was reduced by approximately 36% with a passive exoskeleton during static bending. These outcomes concur with current study, which showed that the longissimus and iliocostalis muscles experienced the highest reduction of 31.38% and 29.76%, respectively.

This study provides insight on how an assistive device can be simulated to aid the agricultural harvesting task. It reveals the ideal location of the assistive device and its basic properties to assist the motion and reduce the MSD risk. For the FFB harvesting task, it was found placing assistive device along the rectus abdominis and iliocostalis is preferred. Placing it along the longissimus is not desirable because it can transmit the load to the rectus abdominis. The basic properties of the exotendon investigated in this study can be replicated by recreating an elastic material or mechanism that exhibits similar resting length and stiffness. Nonetheless, the limitations of this study should be considered. The model’s muscle architectural parameters and the harvesting force assumption might not match those of the real harvesters. Furthermore, this study examined the biomechanical characteristics of the harvester, including joint moment, muscle activation, and muscle force, to determine the muscles that the assistive devices should support. However, other crucial factors such as user experience, durability, compatibility, material, and price of the assistive device should also be investigated when designing the physical wearable assistive technology [8]. It is recommended that a physical prototype exoskeleton to be developed and tested on harvesters. Lastly, the pilot tests on variable resting lengths and stiffness of the assistive device demonstrated that these two parameters could significantly affect the performance of the assistive device. A broader range of resting length and stiffness should be investigated in future.

## Conclusion

Several passive assistive devices were designed, modelled, and integrated into the harvesting musculoskeletal model to augment the back muscles during harvesting. Simulation results demonstrated that the passive assistive devices that assist the rectus abdominis and iliocostalis could aid and support the harvesting task. The longissimus muscle can alleviate the workload by distributing a portion of it to the rectus abdominis. These findings are valuable for the engineer and designer of the assistive device to provide guidelines for which muscles to assist during agricultural harvesting. Future studies to investigate the user experience, durability, compatibility and price of the assistive devices are recommended to provide more in-depth information for designing and developing the optimal physical assistive device for agricultural harvesting.
